# Experiences and narratives of mandatory psychological assessment in Trans and Nonbinary people: An Italian qualitative analysis

**DOI:** 10.1186/s40359-025-02675-3

**Published:** 2025-04-06

**Authors:** Ciro De Vincenzo, Andrea Garolla, Sara Delinna, Laura Pizzolato, Ines Testoni

**Affiliations:** 1 Department of Philosophy, Sociology, Education and Applied Psychology, University of Padova, Via Venezia 14, Padova, Italy; 2 Department of Medicine, University of Padova, Padova, Italy

**Keywords:** Transgender, Nonbinary; gender affirmation pathway, Psychological assessment

## Abstract

**Background:**

This study adopts a qualitative methodological framework to investigate the overall experiences of institutional gender affirmation pathway (GAP) with a focus on the narratives of mandatory psychological assessment in Trans and Nonbinary (TNB) people in Italy, who face daily discrimination, social prejudice, episodes of violence, and victimization. In the Italian context, psychological assessment is a prerequisite to receive a diagnosis of gender dysphoria, which is essential to access hormone therapy, surgical interventions, and legal change of name and gender marker.

**Methods:**

The study employed a qualitative methodological design, since it allows a deep and flexible exploration of participants’ experiences and perspectives. Specifically, the data collection technique consisted of semi-structured interviews with *N* = 21 participants. The corpus of data was analyzed consistently with thematic analysis and reflexive thematic analytic approach, for identifying, analysing and reporting patterns in data, allowing the corpus of data to be examined in terms of their principal themes, using both theory-driven (top-down) and bottom-up analytical strategies.

**Results:**

Through the reflexive thematic approach, we generated two themes, further divided into sub-themes. The first theme is “Being trans + in our society” and it is composed by the following sub-themes: “personal experiences of identity affirmation”; “minority stress and transphobia”; “supportive familiar and working contexts”, “institutional gender affirmation process”; “resilience and self-determination”. The second theme, “Experiences of mandatory psychological assessment” is divided in: “disrespectful experiences”; “affirming and supportive interactions”; “to take better care”. The results highlight how TNB individuals often experience significant stress related to their minority status and face discrimination in healthcare settings. Furthermore, there is a lack of individualization in the approach by healthcare providers and a shortage of knowledge about gender sensible topics among them. This study therefore highlights an insufficient provision of effective psychological support and the need to adopt more inclusive approaches, dismantling the pathologizing dimension of the diagnostic process for TNB people.

**Conclusions:**

It is therefore crucial to promote awareness programs on gender identity issues to foster a more welcoming and informed environment in healthcare settings.

**Trial registration:**

The study has been approved by the Ethics Committee of the University of Padua with unique number D120DC6FDC5DF2694CF281D76B2CDB41 and protocol number 5003.

**Supplementary Information:**

The online version contains supplementary material available at 10.1186/s40359-025-02675-3.

## Introduction

Within the Gender affirmation pathways (GAPs) the mandatory psychological assessment plays a crucial role. Our paper focuses on the experiences and narrative of mandatory psychological assessment in GAPs. Aligning with previous relevant reflection in the field [1–2], we avoid basic definition of trans and gender-diverse people. For the purposes of our research, transgender is an umbrella term that encompasses a diverse range of individuals that embraces incredibly diverse experiences. This includes genderqueer, gender fluid, nonbinary people, trans men and trans women, whereas the term cisgender refers to people whose gender identity aligns with their assigned gender at birth. Since not all nonbinary people identify as transgender, we will adopt the expression Trans and Nonbinary (TNB) when referring to the general population to ensure inclusivity, whereas nonbinary will be used when referring specifically to nonbinary individuals and trans men and trans women will be used when referring exclusively to these groups [3–5].

Historically, TNB identities have been pathologized alongside sexual minorities, beginning with 19th-century sexology’s classification of “inverted” individuals and continuing through mid-20th-century [6–7]. This has led to their classification as psychiatric diagnoses in ICD-9 and DSM-III [8], legitimizing stigmatizing and inappropriate procedures [9–10]. Health and mental health professional communities have played a critical role, adopting approaches which have resulted in the pathologization of non-hegemonic gender expressions through transnormative lenses and restriction of self-determination for TNB individuals [11–17]. Notably, the shift in DSM-5 from “Gender Identity Disorder” to “Gender Dysphoria” has outlined a less stigmatizing position by focusing on the subjective distress that may arise from gender incongruence. However, the diagnosis still imposes normative definitions of trans and nonbinary experiences, perpetuating trauma and discrimination [18–23]. Similarly, ICD-11 represents progress by reclassifying gender incongruence under sexual health conditions rather than mental disorders, reflecting contemporary knowledge that diverse gender identities are not indicative of mental illness. This change mitigates stigma, yet TNB individuals continue to experience discrimination, victimization, and barriers to care [24–18]. The pathologization of non-hegemonic gender expressions results from a transnormative view of diagnosis, while medical and psychological affirmative supportive procedures show positive outcomes for both adults and adolescents [25–32].

The Informed Consent Model (ICM) offers an alternative approach, allowing TNB clients to access hormone treatments and surgeries without mandatory mental health referrals [33–36]. By eliminating the requirement to demonstrate distress or impairment, the ICM emphasizes autonomy, requiring only the cognitive capacity to make informed healthcare decisions [37–44]. Psychologists, under this model, shift their focus from diagnosis to supporting clients in exploring and affirming their gender identity, addressing internalized transphobia, and navigating their coming out processes [33]. This approach fosters trust between clients and providers, enabling TNB individuals to express their needs without fear of stigma or outdated diagnostic frameworks. The ICM also aligns with patient-centered care by prioritizing individual autonomy and ensuring that healthcare procedures are tailored to the client’s informed choices. Studies show that ICM is associated with improved well-being and a stronger sense of belonging for TNB individuals [45–46]. As the debate around TNB healthcare evolves, the role of mental health professionals must adapt to prioritize empowerment and affirm the validity of diverse gender experiences.

### The gender affirmation pathway (GAP) in Italy

Some TNB individuals may choose to initiate a gender affirmation pathway (GAP), which can involve changes to the body through the use of hormones, surgical interventions, and changing their name to live in accordance with their desired gender identity [47–48]. Access to psychological, social, and medical services for gender affirmation, as well as appropriate procedures, aim to improve the psychological and physical well-being of transgender individuals [49–50], although it is not the option for everyone [51–52]. As previous research showed [10], GAPs still fail to provide tailored approaches to unique needs, granting clients the opportunity to follow a personalized pathway tailored to their desires. In Italy, Law No. 164/1982, titled *Norms regarding the rectification of the attribution of sex*, regulates the GAP process, allowing TNB to undergo medical and surgical procedures and to change their name and gender marker, subject to approval by the relevant court [53]. Psychological counseling or assessment has now become standard practice for TNB thanks to Article 2 of the law, which states that the judge may request counseling in order to “ascertain the psychosexual condition of the person concerned.” Furthermore, the diagnosis of GD or gender incongruence plays a significant role in countries, including Italy, where meeting the criterion for diagnosis is necessary to access surgical and/or hormonal interventions through the Public Health System [11, 52]. In Italy, gender affirmation involves a medico-legal process where individuals must undergo medical assessments, often requiring evidence of gender dysphoria and surgical intervention, to legally change their gender on official documents. This system has been criticized for pathologizing gender identity, as it relies on a binary medical framework that complicates self-determination for transgender individuals. However, recent legal progress has challenged this, notably in a 2015 court ruling that allowed for gender marker changes without requiring surgical sterilization. While legal advancements have been made, the process still reflects a complex balance between medical requirements and individual rights, highlighting ongoing challenges for transgender individuals in achieving full citizenship [54]. According to ONIG (Osservatorio Nazionale sull’Identità di Genere) guidelines, which are used in some centers, psychological counseling in Italy should last for at least six months prior to hormonal treatments and serves both diagnostic and supportive purposes. However, TNB individuals have reported in several studies that many mental health practitioners lack basic knowledge and skills to work with this population [54–57]. In contrast, the World Professional Association for Transgender Health (WPATH) guidelines, outlined in the Standards of Care, emphasize a flexible, patient-centered approach, advocating for the removal of restrictive requirements like the “Real Life Test” and prioritizing informed consent [58]. Finally, In Italy, TNB individuals often access psychological assessments through associations that specialize in gender-affirming care. These associations, which operate independently or in collaboration with healthcare providers, play a crucial role in offering psychological support. Given the limitations of the public healthcare system in providing specialized gender-affirming services, these associations serve as key resources for TNB individuals seeking support.

### Implications of mandatory psychological assessment

Within the GAP, psychologists often shape their counselling and assessments of TNB individuals based on personal assumptions influenced by transphobic narratives, reinforcing gender binarism and disregarding scientific knowledge [59–60]. Mandatory psychological sessions, often perceived as involuntary treatment, can undermine trust, lead to hormone acquisition on the black market, and encourage clients to present “perfect” narratives to meet professionals’ expectations shaped by systemic transnormativity [52, 55, 61–63]. These expectations stem from systemic issues, such as gaps in university curricula and societal policies, rather than individual professional biases. TNB clients report experiencing assumptions about genital procedures, misgendering, pathologization, and judgments about body types, leading to feelings of guilt, objectification, and invalidation [64–66]. Such inadequacies exacerbate mental health symptoms, reduce satisfaction with care, and deter future help-seeking [64]. Conversely, psychologists can play a pivotal role in helping clients navigate family dynamics, stigma, and minority stress, while exploring gender identity and roles [67]. Nonbinary individuals face unique challenges, including identity erasure, microaggressions, and systemic binarism in institutional pathways, resulting in longer waiting times and additional barriers [11, 68–69]. Discrimination and violence remain pervasive for TNB individuals. Studies reveal high rates of verbal, physical, and sexual abuse among transgender individuals in Italy, alongside widespread workplace and healthcare discrimination [70–72]. Similarly, European data highlight that over half of TNB individuals experience harassment or discrimination, with significant barriers in employment and healthcare [72].

Based on the cited literature, the present research aims to investigate the overall experiences and narratives of TNB people’s mandatory psychological assessment. In doing so, it seeks to expand on previous findings in the Italian context by providing first-person centered nuanced perspectives.

## The present study 

### Materials and methods

To achieve our objectives, we adopted a qualitative methodological framework employing reflexive thematic analysis of semi-structured interviews. Qualitative methodology is particularly advantageous in social and psychological studies as it allows exploration of participants’ experiences from their unique perspective [73], supporting a flexible collection and analyses of data. Qualitative approaches are idiographic, as they foster the integration between researchers’ and participants’ perspectives and aim at collecting thick descriptions of participants’ experiences [74].

### Participants

The study involved twenty-one participants. In order to be included, each participant should have had at least six months’ experience of psychological session in assessment context. Six participants were transgender women (29%), thirteen were transgender men (61%) and two nonbinary people (10%) who used neuter or masculine pronouns. The participants were aged between 17 and 45 years old (average = 26.42; SD = 7.97), half of which were students (3 high school students, 7 college students). None of the participants had kids and only one of the participants was married. Furthermore, 4 people (19%) reported having a high school diploma, 11 (52%) had a high school diploma, and 6 (29%) had a bachelor’s degree. Psychological assessment was mainly carried out by associations for 16 people (76%), mainly in Veneto (10 people), Piedmont (3) and Emilia Romagna (2). Two people (10%) carried out the pathway by turning to the public service, one Ulss from Veneto and one Asp from Sicily, respectively. Only three people (14%) carried out the GAP process privately (1 in Trentino and 2 in Veneto). To ensure privacy, fictitious names were used (see Table 1).

**Table 1:** Participants’ demographic characteristics

Fictitious names	Age	Degree	Employment	Region of Gender Affirmation Pathways
Grace	22	Graduate	Student	Veneto
Gus	22	Graduate	Student	Veneto
Matteo	17	Middle school diploma	Student/Worker	Sicily
Simone	27	High school diploma	Student/Worker	Emilia Romagna
Daniel	25	High school diploma	Warehouse worker	Veneto
Max	24	High school diploma	Student/Worker	Piedmont
Manuel	27	High school diploma	Worker	Veneto
Giovanni	25	Graduate	Student/Worker	Piedmont
Alice	42	High school diploma	Worker	Veneto
Martino	24	Graduate	Student	Trentino
Salvatore	30	Graduate	Student	Emilia Romagna
Francesca	40	High school diploma	Unemployed	Emilia Romagna
Wilma	35	High school diploma	Unemployed	Veneto
Adam	21	High school diploma	Worker	Veneto
Giulia	45	High school diploma	Worker	Veneto
Elliot	18	High school diploma	Student	Veneto
Riccardo	28	High school diploma	Student/Worker	Veneto
Jonathan	19	High school diploma	Student	Piemonte
Giorgio	19	High school diploma	Student/Worker	Campania
Dalia	19	High school diploma	Worker	Veneto
Axel	26	High school diploma	Worker	Veneto

### Data collection

We conducted semi-structured interviews to understand participants’ viewpoints, enabling in-depth exploration of their experiences and attitudes [75]. We realized the interviews between December 2022 and February 2023. Participants were recruited through associations, trans self-administered groups, and collaboration with an endocrinologist at Padua hospital. The mandatory psychological assessment pathway discussed by participants was in no way connected to the endocrinological pathway and the endocrinologist does not collaborate with the professionals who conduct the mandatory assessment mentioned by the participants. Each participant received an informed consent form detailing the research objectives which they signed prior to the interview. The consent form included permission for audio recording and for publication, ensuring anonymity by the adoption of pseudonyms. The interviews, lasting approximately 60 min each, were mostly conducted via video conferencing platforms (such as Zoom or Google Meet), while two meetings were held face-to-face.

We provide the Semi-Structured Interview Model in Appendix A (see Appendix A). The semi-structured interview explored various themes, including: emotions and experiences linked to psychological assessment; coming out and potential changes in relationships; possible parental bereavement management; emotions connected to psychological experiences, gained resources and instruments; relationship with the mental health field; desires and ideas related to the gender-affirming care. Some examples of questions were “How did the idea of starting psychological sessions make you feel? Do you think it helped you?”, “How did your parents react when you came out as transgender? Do you think psychological assessment and counselling helped you to navigate the new relationship with them?” and “What tools did psychological work leave you with? What would be the ideal GAP for you and why?”.

Participants voluntarily took part in the study and did not receive any reimbursement. The study has been approved by the Ethics Committee of the University of Padua with unique number D120DC6FDC5DF2694CF281D76B2CDB41 and protocol number 5003 and has been performed in accordance with the ethical standards as laid down in the 1964 Declaration of Helsinki and its later amendments or comparable ethical standards.

### Data analysis

We transcribed all the audio recordings verbatim and were analyzed according to thematic analysis and its subsequent improvements within a reflexive frame. Thematic analysis is a method for identifying, analyzing and reporting patterns in data, allowing sources to be examined in terms of their principal themes, using both theory-driven (top-down) and bottom-up or grounded modalities. The analysis of the text followed the six main phases outlined by Braun and Clarke [76]: preparatory organization; generation of categories or themes; coding data; testing emerging understanding; searching for alternative explanations; and writing up the report. As consistent with further developments of thematic analysis, i.e., Reflexive Thematic Analysis [77–79], the reflective dimension, that is the ongoing iterative and abductive process of combining bottom-up and top-down insights, is fundamental in the identification of themes. The software Atlas.ti was used solely as a tool for organizing the codes that were created manually.

Thus, two members of the research team independently coded all the data and met on a weekly basis with the other members to justify their coding choices. Eventually, the disputes were resolved but a third expert helped to combine the empirical data with pre-existing knowledge on the topic. The final codebook serves as the basis to develop primary categories or sub-themes, which function as intermediate semantic structures between codes and themes. Finally, the whole team– working on the codebook and the categories– meet on a regular basis to identify key structuring themes. Final themes were checked back with codes and participants’ words to assess their hermeneutic potential.

### Positionality statement

All members of the research team had prior experience in conducting research or working with TNB people. We also participate in scientific and professional networks addressing TNB people’s challenges from a critical interdisciplinary perspective. Furthermore, we are involved in movements advocating for civil rights and social justice for TNB people. Our professional expertise, civic engagement, and priori experiences have undoubtedly shaped our research design. We recognize that our positionality is shaped not only by our expertise but also by our privileges, identities, and potential biases. While some of us belong to the TNB community, others identify as cisgender, which necessarily impacts our perspectives and the ways in which we relate to participants0 experiences. As researchers embedded in social psychology, a field that has historically played a gatekeeping role in TNB individuals’ access to care and recognition, we acknowledge the ethical responsibility of approaching our work with critical awareness. The history of psychology includes pathologizing frameworks that have contributed to the marginalization of gender-diverse individuals. This recognition informs our commitment to conducting research that does not reinforce historical power imbalances but rather amplifies TNB voices in an affirming and non-exploitative manner. Moreover, we actively engaged for an epistemological shift in psychological research and application toward critical, intersectional, indigenous theories [80] In terms of methodological design, we opted for a qualitative framework. We believe that TNBs’ voices and lived experiences have their own epistemological place in the advancement of scientific understanding and development of nuanced, person-centered psychological support. Doing qualitative research becomes a social constructive tool to reduce experiential distance, familiarize with the uniqueness of personal perspectives, and materialize a critical dialogue who might end challenging disciplinary pre-assumptions and requiring a more dialogic and participatory systemic adaptation. Moreover, our experiences proved invaluable in data collection, for gaining privileged access to potential participants and, most importantly, served as a reliable foundation for building trust during the interviews. Indeed, individuals and groups who experience marginalization or discrimination might exercise a relational diffidence in research context, if not decide to not participate (as we experienced in other intersectional settings [81–82]. Our experiences suggest that these are not only personal attitudes; rather, it is a proactive, empowering performance of feelings of isolation, acted toward social actors and their responsibility. Our background helped us in creating an allied atmosphere during interviews, not risking transforming qualitative research into an exploitative experience for participants or into micro epistemological violence, where participants’ words are merely considered as data to be processed. In terms of data analysis, our background enhanced the ability to contextualize participants’ accounts and experiences within broader sociopolitical frameworks, acknowledging the profound intersection between psychological experiences and context [1]. Finally, we also critically reflected on the role of diagnostic frameworks, particularly the concept of gender dysphoria. While gender dysphoria remains a central construct in medical and psychological discourses, we acknowledge ongoing debates regarding its necessity, pathologization, and implications for access to care. Our approach to analysis was informed by an awareness of these tensions, allowing us to situate participants’ narratives within broader sociopolitical and institutional contexts rather than treating them as isolated clinical phenomena. By embracing a reflexive stance, we aim to foreground the agency and diverse perspectives of TNB individuals, moving beyond deficit-based models toward frameworks that recognize resilience, self-determination, and systemic inequities.

## Results

**Fig. 1:**
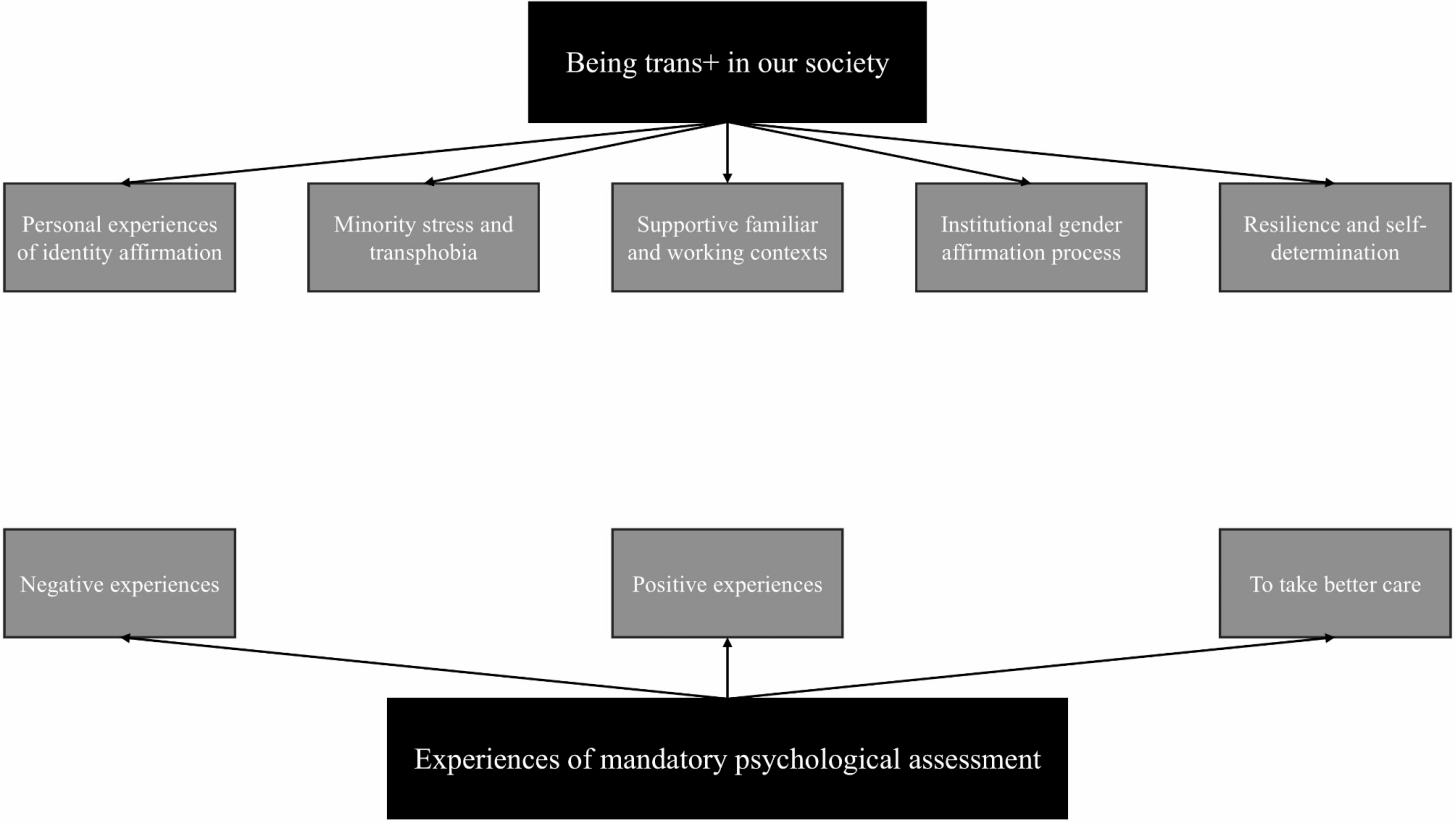
The generated themes and sub-themes

We generated two themes, further divided into sub-themes. The first theme is “Being trans + in our society” and it is composed by the following sub-themes: “personal experiences of identity affirmation”; “minority stress and transphobia”; “supportive familiar and working contexts”, “institutional gender affirmation process”; “resilience and self-determination”. The second theme, “Experiences of mandatory psychological assessment” is divided in: “disrespectful experiences”; “affirmative and supporting interactions”; “to take better care” (see Fig. 1).

### First theme: being trans + in our society

This theme highlights how GAPs are not only an individual experience but are profoundly influenced by relationships with family, society, and workplace contexts. Through its sub-themes, this topic conveys the multidimensionality and multi-contextuality of gender affirmation experiences, traversing the personal sphere (“personal experiences of identity affirmation”), exposure to minority stress and transphobia in various settings (“minority stress and transphobia”), the importance of family and workplace support (“supportive familiar and working contexts”), institutional gender affirmation experiences (“institutional gender affirmation process”), and, finally, the themes of resilience and self-determination (“resilience and self-determination”). Thus, the generation of this theme aims to value experiences as lived unfolding taking place at the intersection between several backgrounds. As will be shown later, such an intertwined background constitutes the ground of the psychological encounter.

### Personal experiences of identity affirmation

Participants shared their journeys of self-recognition and acceptance, emphasizing the deep personal nature of gender affirmation. This process often involved grappling with feelings of dysphoria, self-discovery, and the eventual necessity of taking steps toward alignment with their true selves. One of the first topics discussed and addressed spontaneously by participants during interviews was gender incongruence. Several individuals recalled various childhood experiences and the sensation of dysphoria, which later became clearer and undeniable as they grew up, eventually becoming unbearable. Matteo, a 17-year-old student, shared his experience:

In 2020 I finally accepted this part of me, it is not like I did not know before, but I felt dysphoria for the upper body, and, like, I never went to the sea before that year.

The gap between self-consciousness and the beginning of a journey is filled with fears and uncertainties about what to do, where and how. Each journey is therefore very personal, but what these people have in common is that they are obliged to go to public or private health facilities in order to get gender-affirming care, and they often encounter many barriers.

Francesca, a 40-year-old unemployed woman, shares:

I have always felt like a woman, since I was a little girl. I always played, and then it’s relative, with dolls, I always wanted feminine toys, I dressed as a woman, it’s always been like that.

Similarly, Wilma, a 35-year-old unemployed woman living with her partner, says:

I remember that my being was already showing when I was little, with a preference for certain things over others. I was very drawn to androgynous figures […] I have always hated my legal name and couldn’t understand why, but I’ve always felt it wasn’t mine. Even looking at myself in the mirror, I saw something that didn’t make sense.

Both Francesca and Wilma’s reflections highlight the deeply ingrained and early experiences of gender identity. Their accounts emphasize a sense of self that transcends societal gender norms, showing that identity can be both personal and complex, often shaped long before one can fully articulate it. These statements also touch on the discomfort or disconnect felt when one’s external presentation doesn’t align with their inner sense of identity, something that resonates with many who experience gender dysphoria or struggle with societal expectations of gender.

### Minority stress and transphobia

Participants reported facing pervasive minority stress and transphobia across societal, healthcare, and workplace contexts. They described being “looked down upon” (Giulia, 45 years) feeling compelled to work “twice as hard as others” and experiencing direct discrimination from professionals and peers alike. Wilma shared: “The society you live in makes you sick. You have to work twice as hard as everyone else just to be seen”. Always Giulia (45 years old):

I need to sort out work because, as of now, I have chosen to predominantly live my life as a male to secure the financial aspect. Unfortunately, without the financial aspect, I would not be able to move forward, with appointments, psychologists, and everything else that comes with it.

Francesca (40 years old) spoke about this impossibility of postponing anymore her GAP, saying:

“In my various experiences, I also tried to be male because, for society—or rather, for my family—I had to be that way. Even though it was not a problem for them, at the same time, I was born male and had to be male. So, I tried to do it, but it was not what I felt”.

Axel, 26 years old, recounts that his father said he needed time to process the situation but assured him that he would always love him. However, he adds: “Things took a bit of a downturn because there were comments like, ‘I will never be able to see you as a man; you are too effeminate.’ […] Once, he told me, ‘In my opinion, sooner or later, you will realize you have made a mistake’”. Eventually, his father asked him to leave home when he started hormone therapy, showing how transnormative tropes are acted by family members as well as societal structures and are not only limited to professionals. Regarding health care settings, Max, a 24-year-old student and artist, told us:

When I began my journey, seeing a psychiatrist was still mandatory […] and the psychiatrist I saw at the Association told me straight in the face, during my second session, that being transgender is not normal. […] I was also getting pressure for the interventions I already said I did not want to do.

### Supportive familiar and working contexts

While many participants reported negative societal reactions, some highlighted moments of support and understanding from family, friends, and employers. These supportive environments often played a vital role in enabling their affirmation. Adam (21 years old), for example, does not have to use his deadname at work^1^, and Riccardo (28 years old), whose “boss asked me what name and pronouns I wanted him and my coworkers to use. They were all supportive”. Relationships with friends and family are a vital part of our lives and are too often at risk when TNB people decide to come out. Family members have shown various reactions: there has been fear of others’ prejudices, a strong sense of confusion, and difficulty in understanding and accepting the situation, which in some cases have led to distance and complications in family relationships. At the same time, Giovanni (25 years old) has also shed light on an important issue, that of parental mourning, saying that he has had the support of his mother:

I still appreciated the fact that, for example, at Agedo (Association of Parents of Homosexuals)^2^, many parents have this thing that they say they experience their child’s gender affirmation pathway as a mourning. My mother says she does not understand it because she had never created an image of me in her head, of how I should have been […] so she says that I am still the same person to her.

There has been an improvement in communication and the quality of both familial and friendly relationships after coming out, which has manifested in some cases greater closeness, openness, and constructive dialogues. Their presence has been crucial in accompanying and supporting them throughout the GAP process, as Martino (24 years old) asserts:

Being surrounded by people who have loved me, still love me very much, and without ever asking for anything in return, in the sense of beautiful human bonds that have made evolving as a person extremely safe.

### Institutional gender affirmation process

Participants expressed frustrations with institutional barriers, such as prolonged waiting times, mandatory conformity to specific narratives, and limited choice of professionals. These challenges often compounded the difficulty of navigating gender affirmation processes. Francesca (40 years old) shared feeling obligated to conform to societal expectations: “I had to wear a bra and present myself the way society wanted in order to continue my journey”. Giulia (45 years) shared her experience with the first endocrinologist she consulted after her psychological journey:

“The endocrinologist bombarded me with a series of questions. I thought maybe it was to test me, to say, ‘Look, this is a real change; it is not like taking an aspirin or just any pill.’ […] As I was walking back, I kept thinking, ‘Should I do it? Should I not do it? Should I do it? Should I not do it? Should I do it? Should I not do it?’ and then I said to myself, ‘I will not do it’”.

Adam (21 years old) compared his journey to that of a French friend who, just a couple of months after starting testosterone, was able to have his first consultation for surgical procedures, whereas Adam has to wait 12 months of RLE. He states:

The fact that here in Italy you need a judge’s approval for changing documents or undergoing surgeries weighs on me a bit. Because you, as an external person who only sees me on that one day—you are not my psychologist, you are not my endocrinologist—why should you, as an external person, decide what I can or cannot do?

### Resilience and self-determination

Despite the numerous obstacles, participants demonstrated resilience and determination to navigate their affirmation journeys. This process often led to personal growth, enhanced self-awareness, and improved relationships. Some people also spoke about their pride and self-esteem. Matteo (17 years old), in particular, describes himself as a calm person: “I have nothing against myself because I also have a lot of self-esteem; I like my personality,” he says, adding that he has no regrets and feels good about himself. Adam (21 years old) also shares: “I am comfortable with who I am,” and expresses happiness at having realized he is a transgender person. Daniel (25 years old) describes himself as: “A very simple and very shy person, which is why it was a bit difficult at first to accept this journey.” Other people, like Giulia, a 45-year-old factory worker, do not feel the need to completely discard their past. She says:

“Forty-five years of Giulio can be felt, they are visible, and that is fine; I am at peace with this, I carry it with me, I do not throw anything away. Many people throw it all away, and I see them truly suffering. You cannot just throw it away—how can you? I understand the suffering and all that, but I cannot; I keep this”.

According to Axel (26 years old) people should often focus on the positive aspects of themselves. He shares:

“Sometimes we wallow a bit […] in our inner world and stay so deep inside ourselves that we almost forget the entire external world, including what others might actually feel, and what we might evoke in them”. He adds:

“Wanting to be a better version of ourselves at all costs usually only leads to frustration because […] all you do is evaluate yourself, constantly striving to achieve something that may not even be attainable, or that is perhaps just an ideal in your head, completely detached from reality. Maybe we should simply try to accept ourselves more for who we are, forgive ourselves every now and then, and perhaps take a bit more notice of the people around us”.

### Second theme: experiences of mandatory psychological assessment

The second theme gathers the narratives and experiences of mandatory psychological assessment, whether it “disrespectful experiences” or “affirming and supportive interactions”. Notably, these sub-themes are not mutually exclusive as they would refer to a strict experiential division; on the contrary participants described both negative and positive interactions within the same experience. Moreover, the theme has a third sub-theme, “to take better care”, reflecting participants’ perspectives and ideas for a better organization of institutional GAP, included the mandatory psychological assessment.

### Disrespectful experiences

This sub-theme highlights participants’ negative experiences during the mandatory psychological assessment, necessary to diagnose gender incongruence and proceed with GAP. For many participants, the psychological assessment’s perceived purpose often revolved more around obtaining access to hormones than the expectation of receiving meaningful support. Several participants reported feelings of anxiety, agitation and fear. For some, these emotions were linked to concerns about needing to “prove myself to the psychologist” (Gus, 22 years old) and “feeling obliged to express myself according to psychologist’s expectations” (Max, 24 years old). While not all experienced anxiety at the beginning, many reported dissatisfactions stemming from gatekeeping practices, prolonged timelines, and feeling compelled to adhere to a specific narrative. Salvatore (30 years old), for instance, described his experience as emotionally draining and shared that success depended on telling the therapist what she wanted to hear:

She was unable and tendentially also violent in her approach. I saw it as a necessity, like, it is something I have to do. I felt really bad every time I had to go there, I would brace myself beforehand because I knew I would feel bad. Until at a certain point, I discovered that to make it work, you basically have to tell her what she wants to hear.

Three participants recounted experiences of discrimination, including psychologists misusing their legal names and pronouns. Axel (26 years old) shared an incident where the psychologist dismissed his gender identity, suggesting it was a means of seeking control in his life:

Look, you are not like that, you are just boxing yourself in. It is just that you want to have more control over your life, so you think that maybe if you were a guy, then you would have more.

In cases where therapists facilitated family sessions, participants often reported additional stress due to a lack of sensitivity and professionalism. Simone (27 years old) recalled a session where his mother was reduced to tears after being labeled a “bad mother” for working on Sundays, highlighting a dynamic of blame rather than support.

### Affirming and supporting interactions

During the psychological mandatory assessment, participants did not solely experience aggressive interactions. On contrary, the relationship they formed with the professionals also led to live empowering and supportive feelings. This sub-theme gathers participants’ positive experiences during the mandatory psychological pathway, highlighting its role in fostering self-discovery, emotional growth, and relational improvements. Manuel’s (27 years old) experience reports:

It started with gaining clarity about why I felt male and did not see myself as coherent in the mirror. Over time, it became about building my identity and shaping how I interact with the world as a male, which is vastly different from navigating the world as a female.

Many participants reported acquiring tools for self-determination and personal growth that extended beyond the gender affirmation process. They described feeling relieved from the burden of others’ judgments, gaining deeper insight into interpersonal dynamics, and enhancing their emotional well-being. Alice (42 years old) shared: “In the deepest period of depression, I felt like a crying child unable to stand. After the sessions, that child grew up, took control, and helped me move forward”.

The psychological journey also improved participants’ relationships, fostering greater openness and assertiveness in their interactions. Elliot, 18 years old, described the changes in managing his emotions and relationships:

At first, I would react impulsively to anything triggering dysphoria, getting angry or upset. Now, I pause to think and consider if it is worth reacting. Even when someone makes a mistake, I take a deep breath and calmly explain the situation.

### To take better care

The last theme collects criticisms regarding the gender affirmation process, as participants have expressed opinions regarding their ideal path. Only two of them, Giulia (45 years) and Francesca (40 years old), stated that their ideal path was the one they underwent. Others, however, expressed the need to introduce the model of informed consent in Italy, as stated by Max (24 years old):

In my opinion, psychotherapy should be free, accessible, and not mandatory […] and changes to legal documents should be achievable through a simple form at the registry office, with various interventions permitted under public health services following the signing of an informed consent form, signed by the individual taking responsibility for altering healthy organs.

In line with this, some participants advocate for a simplified bureaucratic process and a more individualized approach to care, beyond a protocol with fixed timelines and costs. They propose an approach that allows for open discussion with field-trained professionals and an in-depth exploration of the risks and psychological and endocrinological effects of hormone therapy for those undergoing treatment.

Regarding this, Giovanni (25 years old) states:

In my opinion, we need to have a less rigid protocol and pay attention to the individual because there are a thousand trans people with a thousand different stories. There are truly people who need support for that because they live in a strong state of discrimination. We also need to accept that many problems related to transgenderism are societal, and as a result, there are individual psychological issues, but it is not the same for everyone.

Manuel, who is 27 years old, states:

“It would be helpful to remove the time requirements for documents and medical procedures because I believe that when you undergo a well-structured process, you can still achieve a certain level of awareness. It is true that hormone therapy changes you, and probably—speaking about my own case—removing my chest immediately is the greatest desire I have right now. I understand that, in terms of impact, it might be very intense for a person, but the desire to remove this part is just as strong. So yes, I would like there not to be a minimum one-year requirement for hormone therapy”.

## Discussion

This study has provided a comprehensive exploration of the complexities surrounding the GAP for TNB individuals in Italy, specifically focusing on their experiences and narratives of the mandatory psychological assessment. The findings offered a nuanced understanding of the multifaceted challenges and opportunities within this pathway, highlighting the interplay between personal, relational, and systemic factors. Thus, the study also revealed that gender affirmation is deeply embedded in social, familial, and workplace contexts, rather than being solely a medical pathway. This is consistent with an ecological perspective, offering targeted understandings and interventions toward the multilayered experiences of TNB individuals [83]. Rather than approaching psychological assessments and the need for psychological support as separated by the overall TNB’s experiences, the thematic organization aimed at highlighting the complex intersection with broader instances in TNB lives. That is relevant for mental health professionals working in the field, as it opens a window to the background and everyday contextual experiences of TNB individuals, that is the lived experiences they bring. Overall, this research found that TNB people are still exposed to several instances of minority stress and discrimination in social and health care settings, confirming previous evidence regarding the Italian sample [28, 55, 71–72]. Finally, our study also found instances of resilience and resistance acted through different ecosystems as well as realized in several interactive situations [84], which also help to shed a better light on micfroaffirmations experienced in everyday life [85].

Moreover, some further reflections can be developed. Participants shared deeply personal narratives of self-recognition and identity affirmation, underscoring the importance of autonomy in the gender affirmation process. These accounts align with existing literature on the value of self-determination in fostering psychological well-being [1–5]. Despite systemic barriers, participants demonstrated remarkable resilience and self-determination. These qualities enabled them to navigate challenging circumstances and achieve personal growth. However, the fear and uncertainty reported by participants, particularly at the beginning of their journeys, highlight the need for supportive procedures that respect individual timelines and needs. Consistent with previous research [11, 66, 69, 72], participants described pervasive minority stress and transphobia across societal and institutional settings as well as from family members. The systemic discrimination they face exacerbates their psychological vulnerability, making the role of healthcare providers critical in mitigating these stressors. On that regard, supportive relationships with family, friends, and employers emerged as key protective and proactive factors. Participants who experienced affirmation from their social networks reported greater resilience and well-being. These findings resonate with previous research [67] highlighting the protective role of affirming relationships. However, the contrast between supportive contexts and discriminatory environments highlights the uneven distribution of affirming experiences, necessitating broader societal change. Aligning with previous research in the Italian context [4, 11, 52], the institutional pathways for gender affirmation in Italy are characterized by rigidity and a lack of individualization. Participants often felt compelled to conform to normative narratives to access care, reflecting a transnormative framework that privileges binary identities.

Rather than being supported in a delicate moment of their existence, people often found a slightly and implicit judgmental environment where their feelings and experiences could not be openly shared. Such a scenario inevitably corroborates and enhances a negative community circuit where one’s own experiences are delegitimized, leading to a secondary socially induced stress. Thus, mental health in these individuals can be at risk due to the harsh environment they live in, and therefore psychologists and psychosocial support can play a fundamental role.

Many participants reported negative experiences during mandatory assessments, describing them as gatekeeping practices that prioritized bureaucratic requirements over meaningful support. This aligns with critiques of transnormativity in healthcare, which imposes restrictive narratives and reinforces binary frameworks [60–63]. Participants also highlighted the emotional toll of these practices, including anxiety and fear of not meeting psychologists’ expectations. Some participants postponed reaching out to health care services until it became inevitable to express how they were feeling to others. As a matter of fact, some of these people started to have internalizing disorders (depression, anxiety, etc.), which interfered with these people’s relationships. As literature suggested [86–39], this study found out that many participants have arrived at their first psychological session experiencing emotions such as anxiety and fear, partly due to the process itself and prejudices surrounding healthcare professionals and their function in such a pathway. This apprehension stems from the fact that these professionals work in the field of mental health and often lack updated knowledge regarding gender identity issues [68]. Half of the participants have reported negative experiences regarding the mandatory psychological assessment or consultation, although some have managed to switch psychologists and find a professional they feel comfortable with. However, this was not possible for everyone, as many individuals sought assistance from public services or associations where the allocation of psychologists could not be changed. This data is concerning as it highlights the limited understanding of gender identity issues within Italian psychology professional sector, as also emphasized in the literature [55]. Associations typically have a very limited psychological team, consisting of three or four individuals, resulting in a lower supply compared to the increasing demand in recent years. Many individuals who have had generally negative experiences, as highlighted in Elder’s research [87], have indeed reported difficulty in discussing themselves and the lack of individualization/personalization in treatment, while stating that they have received bureaucratic support. Furthermore, not only have some individuals experienced discrimination from their psychologists or psychiatrists, despite existing guidelines [88], but they have also felt compelled to uphold a certain narrative to continue their affirmation pathway [67]. Moreover, many individuals were unable to fully express their individuality because support was often solely focused on the GAP, and it was not possible to discuss other aspects of life [83]. That is concerning, as it reinforces a perspective on GAP which seems to overlook its significance and salience with one’s own whole psychological life and experiences. Conversely, those who found psychologists ready to welcome them from the outset completed their journey calmly, without haste, managing to delve into various aspects of themselves. Here seems to lie the differences between gatekeeping and supportive function psychologists might perform in such a setting, that is in their willingness to act and share an openness toward one’s own life as a whole, rather than solely focus, inspired by a medical model, on the affirmation pathway itself. While all individuals were satisfied with the diagnostic aspect, as they received a gender incongruence diagnosis, psychological support was lacking in too many cases. Participants frequently experienced negative feelings due to the lengthy process and the timing of surgical procedures, both of which are crucial pathways for improving mental health in the TNB population [89–90]. Although the literature suggests on one hand that many TNB individuals choose, when possible, to undergo GAP with limited or no use of mental health practitioners [71], and on the other hand, that negative experiences in psychotherapy may hinder future help-seeking [68, 91–92], almost all participants continued with psychological assessment in cases where it had been positive, sought another therapist, or were willing to seek psychological help in the future. Furthermore, in expressing preferences regarding the GAP, many participants have emphasized the need for psychological support, although not mandatory, during the gender affirmation process. This process entails significant changes in the relationship with oneself and with others, sometimes requiring support aimed at finding the best path for that individual [63]. While negative experiences were prevalent, some participants reported positive outcomes, such as personal growth, enhanced self-awareness, and improved relationships. These findings underscore the potential for supportive practices, even within a gatekeeping framework. The concept of microaffirmations provides a useful lens for understanding how small, affirming actions by professionals can foster trust and well-being. The dual role of psychologists as gatekeepers and supportive figures warrants critical reflection. While the gatekeeping role often undermines trust and autonomy, integrating affirming practices can mitigate these tensions and foster more inclusive care. Finally, participants offered valuable suggestions for improving the gender affirmation process, advocating for the adoption of the informed consent model [33–34]. This approach prioritizes patient autonomy and reduces the stress associated with mandatory assessments. Additionally, participants emphasized the need for more personalized care, highlighting the limitations of rigid protocols and the importance of addressing individual needs.

## Conclusion

The findings from our research have highlighted dense experiences and multifaceted narratives. Even though participants expressed personal resilience, narratives of sympathetic familiar and workplace contexts, and positive psychological experiences, overall TNB individuals are still exposed to unsupportive and discriminatory social, institutional, and professional environments. The psychological mandatory assessment is still far from being affirming and supportive of TNB identities, failing to accommodate diverse needs and experiences and does not provide support across various aspects of an individual’s life. Different and multi-level recommendations, involving multiple social and professional agents, could be identified from our findings, strengthening an ecological approach to translational changes and improvements in the field. Practitioners should dismantle gatekeeping roles when providing psychological assessments and build a trustworthy relationship with their clients, clearly defining goals, limits, and boundaries of the diagnosis, presenting it as an institutional requirement rather than an evaluation of authenticity. Along the same line, healthcare organizations and stakeholders should support professionals to become aware of discrimination against TNB individuals by their peers and to promote different approaches aimed at self-affirmation for TNB individuals and understanding of their unique needs. As mental health professionals, there has emerged a necessity to become more aware of discrimination against transgender individuals by colleagues and to support different approaches. These may include, for example, starting to promote awareness programs even in developmental stages, supporting the creation of therapeutic groups to collectively overcome issues related to minority stress, and increasing awareness of these issues within university courses, in order to educate future professionals who are more conscious of their cultural background and any biases it may entail. Furthermore, there is a need to dismantle the pathologizing aspects of the diagnostic process, moving towards a model that emphasizes support, affirmation and self-determination and focuses on each person’s unique experience. Training in this area is scarce in university contexts, which underscores the importance of developing modules in the future that address this topic and enable psychologists to provide adequate support to this population. Apart from a professional and organizational level, also institutions and policymakers should cherish participants’ words and experiences by enriching the possibility to tailor pathways based on individual needs, eliminating gatekeeping, and smoothening or reducing bureaucracy. Finally, relevant context, such as family, workplace, community settings, and university programs should support training and supportive tailored programs to foster trans-affirmative practices and advocating for legislative changes.

The limitations of this research include the lack of heterogeneity among the participants, the majority of whom were from Northern Italy, and all of whom were white. It would indeed be important to understand the differences in the training of psychologists between Northern, Central, and Southern Italy, as well as to explore the experiences of TNB individuals with a migratory background and how aspects of transphobia intersect with racial discrimination. Similarly, it would be important to direct research towards investigating any differences in the approach to the psychological mandatory assessments among adolescents, young adults, adults, and seniors [39]. Additionally, understanding the level of awareness of psychologists regarding their abilities is crucial. Finally, the research only examined two experiences of nonbinary individuals, which often encounter more difficulties in healthcare settings compared to binary transgender individuals.

## Electronic supplementary material

Below is the link to the electronic supplementary material.

Supplementary Material 1

## Data Availability

The datasets generated and/or analysed during the current study are not publicly available due to privacy but are available from the corresponding author on reasonable request.

## Footnotes

1 The name a transgender person was given at birth but no longer uses.

2 Association of parents, relatives, and friends of lesbian, gay, bisexual, trans*, + people.
