# Supplementary material for: Experiences and narratives of mandatory psychological assessment in Trans and Nonbinary people: An Italian qualitative analysis
Source: BMC Psychol. 2025 Apr 6;13:342. doi: 10.1186/s40359-025-02675-3 (PMC11972465; doi:10.1186/s40359-025-02675-3)
Supplement: Supplementary file 1 — Supplementary Material 1 [file 40359_2025_2675_MOESM1_ESM.pdf]

APPENDIX A

Semi-Structured Interview Model

Preliminary: Socio-demographic questions (the gender the interviewee identifies with, the pronouns the interviewee uses, their age, region of origin, number of people in their family, level of education)

1. Can you tell me about yourself and your story?
2. How did the idea of starting psychotherapy make you feel?
3. How did you experience those months? How did you feel?
4. Do you think it helped you? What was its purpose according to you?
5. How did your parents react when you came out as transgender?
6. Has your relationship with them changed?
7. Do you think psychotherapy helped you process the new relationship with them?
8. If yes, how? If no, why not?
9. What tools has psychotherapy given you?
10. How do you feel now when you think back on it?
11. Do you ever think back to those months?
12. If yes, how do you feel?
13. Has your relationship with therapy changed?
14. Have you ever considered going back to therapy?
15. What would be the ideal gender affirmation process for you and why?
